# Ensemble methods of rank-based trees for single sample classification with gene expression profiles

**DOI:** 10.1186/s12967-024-04940-2

**Published:** 2024-02-07

**Authors:** Min Lu, Ruijie Yin, X. Steven Chen

**Affiliations:** 1 Division of Biostatistics, Department of Public Health Sciences, Miller School of Medicine, University of Miami, 1120 NW 14th Street, Miami, FL 33136 USA; 2 Sylvester Comprehensive Cancer Center, Miller School of Medicine, University of Miami, 1475 NW 12th Ave, Miami, FL 33136 USA

**Keywords:** Single sample predictor, Decision tree, Rank discriminant, Ensemble learning, Boosting, Random forest

## Abstract

Building Single Sample Predictors (SSPs) from gene expression profiles presents challenges, notably due to the lack of calibration across diverse gene expression measurement technologies. However, recent research indicates the viability of classifying phenotypes based on the order of expression of multiple genes. Existing SSP methods often rely on Top Scoring Pairs (TSP), which are platform-independent and easy to interpret through the concept of “relative expression reversals”. Nevertheless, TSP methods face limitations in classifying complex patterns involving comparisons of more than two gene expressions. To overcome these constraints, we introduce a novel approach that extends TSP rules by constructing rank-based trees capable of encompassing extensive gene-gene comparisons. This method is bolstered by incorporating two ensemble strategies, boosting and random forest, to mitigate the risk of overfitting. Our implementation of ensemble rank-based trees employs boosting with LogitBoost cost and random forests, addressing both binary and multi-class classification problems. In a comparative analysis across 12 cancer gene expression datasets, our proposed methods demonstrate superior performance over both the k-TSP classifier and nearest template prediction methods. We have further refined our approach to facilitate variable selection and the generation of clear, precise decision rules from rank-based trees, enhancing interpretability. The cumulative evidence from our research underscores the significant potential of ensemble rank-based trees in advancing disease classification via gene expression data, offering a robust, interpretable, and scalable solution. Our software is available at https://CRAN.R-project.org/package=ranktreeEnsemble.

## Introduction

The heterogeneity of cancers necessitates the precise classification of patients into correct cancer subtypes for both prognosis and effective treatment. In the past two decades, the utilization of gene expression profiles has increasingly demonstrated success in identifying cancer subtypes [1–5]. Numerous studies have highlighted the potential of using gene expression profiles for cancer tissue classification, leveraging both statistical and machine learning models. However, these models often encounter challenges in data transformation, normalization, and management of batch effects, which can significantly impact their performance [6–9]. A notable issue is “test set bias”, where predictions for an individual patient vary depending on the patient sample group used in the normalization process, rather than reflecting the patient’s unique characteristics [10].

An emerging alternative for single sample classification is the Single Sample Predictor (SSP) approach [11–15]. This method offers significant advantages, such as the ability to utilize samples from diverse gene expression platforms without the need for calibration. SSPs enable personalized predictions by focusing on the unique attributes and contexts of individual samples, rather than relying on aggregated or generalized trends from larger datasets [16, 17]. Consequently, SSP methods are promising for developing precise and robust classification rules that are effective across various studies and platforms.

Typically, SSP methods utilize either nearest centroids methods [11, 12] or rank statistics of gene pairs [18, 19], the latter often being referred to as Top Scoring Pairs (TSP) based methods [20, 21]. Centroid-based methods classify samples based on proximity to the nearest centroid in feature space, typically using distance metrics like Euclidean distance. Although intuitive and effective in cases with distinct class centroids, they may underperform with overlapping classes or complex class boundaries. Furthermore, these methods were not primarily designed for individual sample concordance, leading to potential inconsistencies in patient-to-molecular subtype assignments [22]. In contrast, TSP methods and their extensions [19, 23–26] offer scalability, interpretability, and robust feature selection. They generate gene rules by comparing expression values within a single sample, thus avoiding normalization with another dataset. However, their classification accuracy has often been suboptimal, limiting their clinical applicability and underscoring the need for more accurate and robust decision models.

In this study, we propose an advancement of TSP methods through the construction of rank-based trees combined with ensemble learning techniques. A single-split tree is analogous to a TSP classifier, and developing deeper trees represents the integration of multiple TSPs for formulating a comprehensive decision rule. To mitigate overfitting, we create multiple trees and ensemble them using techniques such as random forests and gradient boosting, thereby expanding the TSP framework from basic one-to-one gene comparisons to a more complex many-to-one or many-to-many interaction model. Our approach not only enhances the TSP method but also leverages the strengths of ensemble learning. Building upon the work of [27], who demonstrated a basic random forest strategy comparable to the k-TSP method, our paper extends this by employing multi-class trees with class-balanced sampling. This strategy improves computational efficiency and prediction performance. Moreover, we extract interactive ranked gene pairs from our random forest model for added interpretive depth. To maximize predictive power, we meticulously tune and compare various parameters for tree construction and ensemble strategies. Additionally, recognizing the prevalence of noise and redundancy in gene expression data, we implement dimension-reduction techniques. These techniques are crucial for eliminating irrelevant features and isolating the most informative and discriminative patterns, thereby facilitating more efficient analysis and interpretation.

## Methods

### Rank-based trees

**Fig. 1:**
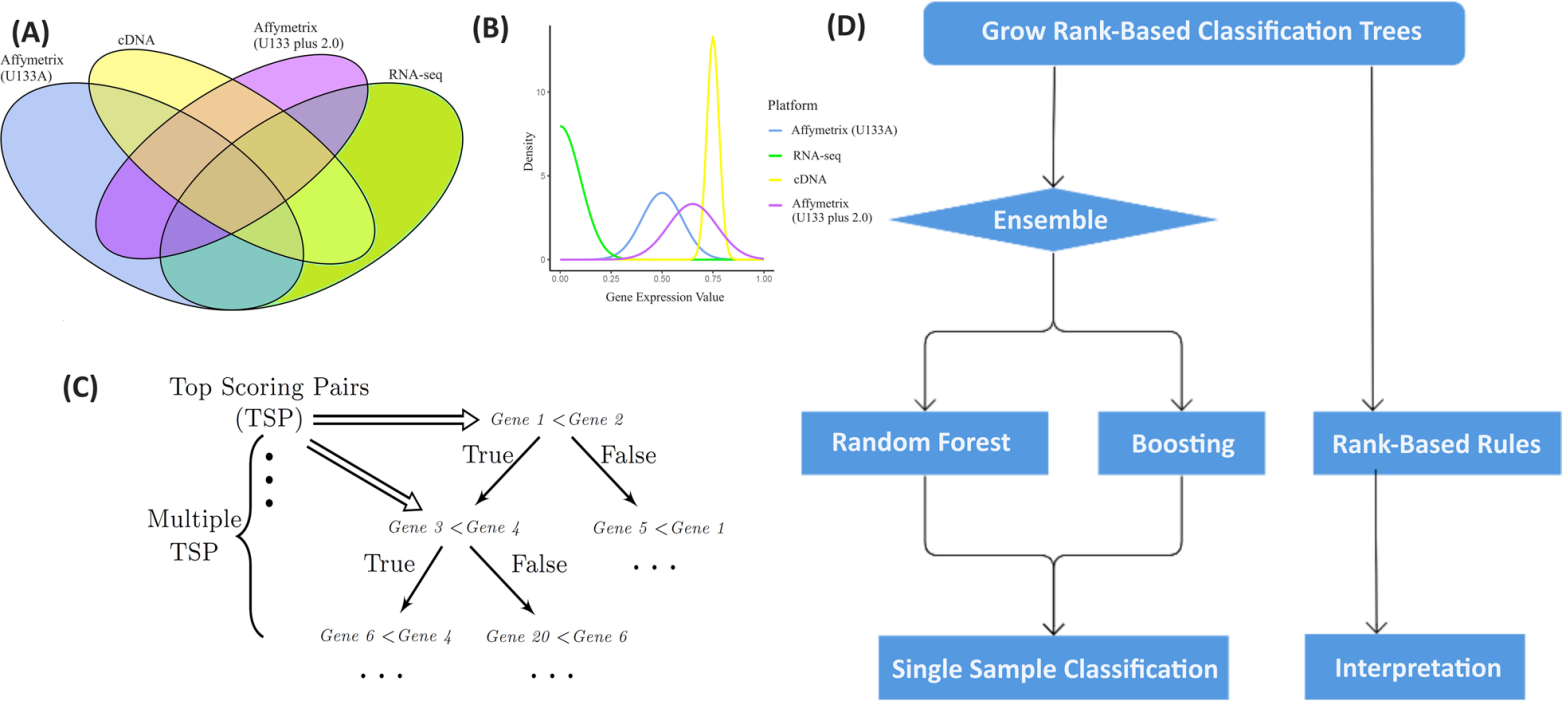
**A** Examples of different platforms to obtain gene expression values. The venn diagram illustrates that certain prediction models are either unavailable or lack predictive accuracy when applied to data from particular platforms. **B** An illustration to show that gene expression values from different platforms are not comparable due to variations in chemistry, quantification, and normalization techniques used by each platform. **C** Illustration of a rank-based tree. **D** The methodological framework utilizing rank-based trees

In this section, we introduce a general framework for rank-based trees using pairwise gene comparisons among a number of gene expressions. Let ${{\textbf {X}}}=(X_1,X_2,\dots ,X_P)$ denote the expression values of *P* genes on an expression matrix, which could be generated from different platforms (see Fig. 1 subfigures A and B for conceptual illustration). Our objective is to use ${{\textbf {X}}}$ to distinguish among *K* phenotypes for the cells in the tissue, denoted as $Y\in \{1,\dots ,K\}$. (Since the boosting algorithm only accommodates binary outcomes, we denote $Y\in \{-1,1\}$ for the boosting case.) A tree classifier is inferred from training data $\mathcal {L}=\{({{\textbf {X}}}^{(1)},Y^{(1)}),\dots ,({{\textbf {X}}}^{(N)},Y^{(N)})\}$, where $({{\textbf {X}}}_i,Y_i)$ are independently distributed. For a given expression vector ${{\textbf {x}}}$, a classifier *h* associates it with a label $h({{\textbf {X}}})\in \{-1,1\}$. We denote the tree predictor of $h({{\textbf {x}}})$ as $h({{\textbf {x}}},\Theta ,\mathcal {L})$, where a parameter vector $\Theta =(\theta _1,\theta _2,\dots ,\theta _T)$ associates the parameter $\theta _t$ with the *t*-th terminal nodes and *T* denotes the total number of terminal nodes.

To grow a rank-based classification tree, the splitting rule can be described as follows. If ${{\textbf {p}}}=(p_1,\dots ,p_K)$ are the class proportions of outcome *Y* for classes 1 through *K*, the Gini index of impurity is defined as

$$\begin{aligned} \phi ({{\textbf {p}}})=\sum _{k=1}^{K}p_k(1-p_k)=1-\sum _{k=1}^{K}p^2_k. \end{aligned}$$

As shown in Fig. 1C, by splitting features recursively into left and right daughter nodes, a tree is grown by minimizing tree impurity. The Gini index split statistic for a split on node *s* on a pair of features $X_i$ and $X_j$ at a given tree node is

$$\begin{aligned} \theta (Y,X_i,X_j,s)=\frac{n_l}{n}\phi ({{\textbf {p}}}_l)+\frac{n_r}{n}\phi ({{\textbf {p}}}_r), \end{aligned}$$

where the subscripts $l=\{X_j\le X_k\}$ and $r=\{X_j> X_k\}$ denote the left and right daughter nodes formed by the split at *s* and $n_l$ and $n_r$ are the sample sizes of the two daughter nodes; $n=n_l+n_r$ is the parent sample size. With some algebra, this is equivalent to maximizing the split statistic

1 $$\begin{aligned} g(Y,X_i,X_j,s)=\frac{1}{n}\sum _{k=1}^{K}\frac{n^2_{k,l}}{n_l}+\frac{1}{n}\sum _{k=1}^{K}\frac{(n_k-n_{k,l})^2}{n-n_l}, \end{aligned}$$

where $n_{k,l}$ is the number of cases of class *k* in the left daughter node and $n_k$ is the number of cases of class *k*; $n=\sum _{k=1}^{K}n_k$ is the total sample size. At tree node *s*, we randomly select a set of candidate features ${{\textbf {X}}}^{(s)}=\{X_{1'},\dots ,X_{Q'}\}$, $Q'\le P$, and the pair of variables with indices $(i_s,j_s)$ will be split if

2 $$\begin{aligned} (X_{i_s},X_{j_s})=\mathop {\mathrm {arg\,max}}\limits _{i,j} g(Y,X_i,X_j,s). \end{aligned}$$

We partition the expression values into a set of gene pairs for constructing splits in the tree nodes and trees are built in a binary fashion: each internal node has an associated splitting rule that uses two predictors, $X_i$ and $X_j$, to assign a observation *k* to either its left or right child nodes, $\{X^{(k)}_i\le X^{(k)}_j\}$ or $\{X^{(k)}_i> X^{(k)}_j\}$. The terminal nodes thus identify a partition of the observation space according to the subdivision defined by a series of splitting rules. For each terminal node *t*, we can arrange the variable indices in pairs $\{(i_1,j_1),\dots ,(i_t,j_t)\}$, $t=1,\dots ,T-1$, such that $\theta _t=\{{{\textbf {x}}}: x_{i_1}<x_{j_1}, \dots , x_{i_t}<x_{j_t}\}$. For a binary outcome $Y\in \{-1,1\}$, we calculate the estimated probability for a given ${{\textbf {x}}}$ as the proportion of class label 1 at the corresponding terminal node $\theta _t$, $p({{\textbf {x}}}) = \mathbb {P}(Y=1|{{\textbf {x}}}\in \theta _t)$ and estimate $\mathbb {E}[Y|{{\textbf {x}}}]$ as $f({{\textbf {x}}})=2p({{\textbf {x}}})-1$. The estimator for a multi-class outcome of *K* labels can be calculated as the proportion of the corresponding class label, $p_k({{\textbf {x}}}) = \mathbb {P}(Y=k|{{\textbf {x}}}\in \theta _t)$ and the tree takes a Bayes classifier $h({{\textbf {x}}})=\mathop {\mathrm {arg\,max}}\limits _{k\in \{1,\dots ,K\}}\mathbb {P}(Y=k|{{\textbf {x}}}\in \theta _s)$.

### Random Rank Forest

The rank-based trees could be of low accuracy with high variance. To prevent overfitting, we first ensemble these trees in a fashion of random forest [28, 29]. As in [28], we define a collection of randomized tree predictors $\{h(\cdot ,\Theta _m,\mathcal {L}), m =1,\dots ,M\}$. We denote the *m*th tree predictor of $h({{\textbf {x}}})$ as $h({{\textbf {x}}},\Theta _m,\mathcal {L})$, $m=1,\dots ,M$, where $\{\Theta _m\}$ are independent identically distributed random quantities encoding the randomization needed for constructing a tree, which are selected prior to grow the tree. These pre-selected parameters are refered to as tuning parameters and discussed in the Discussion section. The tree predictors are combined to form the finite forest estimator of $h({{\textbf {x}}})$ as

3 $$\begin{aligned} \hat{p}_k({{\textbf {x}}})=\hat{P}(Y=k|{{\textbf {x}}})=\frac{1}{M}\sum _{m=1}^{M}\mathbb {1}_{\{h({{\textbf {x}}},\Theta _m,\mathcal {L})=k\}} \end{aligned}$$

and $$h({{\textbf {x}}})=\mathop {\mathrm {arg\,max}}\limits _{k\in \{1,\dots ,K\}}\hat{p}_k({{\textbf {x}}})$$.

Although random forest offers the advantage of achieving high levels of accuracy, the decision rules become extremely complex after averaging the rank based trees, which motivates us to extract information from the blackbox to increase interpretability. Since each terminal node of a tree can be viewed as a classification rule from multiple TSPs, we propose the Algorithm 1 to identify some importance classification rules. Note that each tree in a random forest algorithm is fitted from a bootstrap sample of the original data, leaving approximately 1− 0.632 = 0.368 out-of-sample data for each tree which is called out-of-bag (OOB). This data can be utilized to estimate the prediction performance and obtain an OOB prediction error without the need for an additional cross-validation step to evaluate the prediction error. Here we calculate the OOB prediction error for each terminal node for selecting rules in Algorithm 1.

**Algorithm 1:**
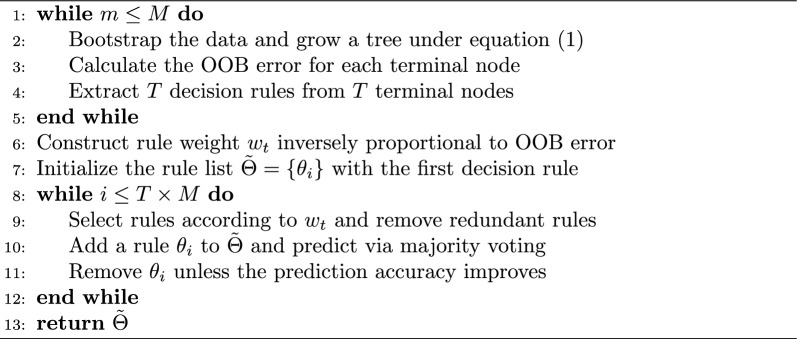
Extracting rules via rank-based trees

Rules from Algorithm 1 are constructed with multiple TSPs so they are high-order classification rules. The classification rule is more interpretable than the classic permutation-based variable importance from random forests and might contribute to biological understanding. In our empirical studies, the top rules tend to be more complex than the simple decision rules from TSP methods, and less number of rules are needed to achieve comparable results as the *k*-TSP method. Although we can aggregate these rules in the fashion of TSP methods, we found that random rank forests always show better prediction performance. Therefore, classification rules only serve the purpose of interpretation, instead of prediction.

### Boosting with the LogitBoost cost

As another ensemble technique, boosting [30, 31] has been used as a powerful tool for classification, especially in high-dimensional settings. As weak learners, random rank trees are ensembled according to a LogitBoost cost function [32] $C(y_i,F({{\textbf {x}}}_i))=\log (1+\exp (-2y_iF({{\textbf {x}}}_i)))$ with $y_i\in \{-1,1\}$, where $F({{\textbf {x}}}_i)=\frac{1}{2}\log (\frac{p({{\textbf {x}}}_i)}{1-p({{\textbf {x}}}_i)})$ and $p({{\textbf {x}}}_i)=\mathbb {P}(y_i|{{\textbf {x}}}_i)$. In each iteration *m*, a regression tree is fit using the negative gradient of $C(y_i,F({{\textbf {x}}}_i))$ as working responses

$$\begin{aligned} z_m({{\textbf {x}}}_i)=-C'(y_i,F({{\textbf {x}}}_i))=\frac{2y_i}{1+exp(2y_iF_{m-1}({{\textbf {x}}}_i))}. \end{aligned}$$

For a tree with *S* terminal nodes, the update uses a refined optimization with unique estimates for each terminal node:

4 $$\begin{aligned} F_m({{\textbf {x}}})=F_{m-1}({{\textbf {x}}})+\lambda \sum _{s=1}^{S}\gamma _{s,m} \mathbb {1}_{\{{{\textbf {x}}}\in \theta _{s,m}\}}, \end{aligned}$$

where

$$\begin{aligned} \gamma _{s,m}= & {} \mathop {\mathrm {arg\,min}}\limits _{\gamma }\sum _{{{\textbf {x}}}_i\in \theta _{s,m}}C(y_i,F_{m-1}({{\textbf {x}}}_i)+\gamma )\\= & {} \frac{\sum _{{{\textbf {x}}}_i\in \theta _{s,m}}z_m({{\textbf {x}}}_i)}{\sum _{{{\textbf {x}}}_i\in \theta _{s,m}}|z_m({{\textbf {x}}}_i)|(2-|z_m({{\textbf {x}}}_i)|)}. \end{aligned}$$

Note that unlike Eq. (1) for a classification tree, the splitting rule for partition $\theta _{s,m}$ is similar to a regression tree [33], which maximizes

5 $$\begin{aligned} g'(z_m({{\textbf {x}}}_i),X_i,X_j,s)=-\sum _{{{\textbf {x}}}_i\in \theta _{s_l}}[z_m({{\textbf {x}}}_i)-\bar{z}_l]^2-\sum _{{{\textbf {x}}}_i\in \theta _{s_r}}[z_m({{\textbf {x}}}_i)-\bar{z}_r]^2, \end{aligned}$$

where the subscripts $l=\{X_j\le X_k\}$ and $r=\{X_j> X_k\}$ denote the left and right daughter nodes for *s*; $\bar{z}_l$ and $\bar{z}_r$ denote the average of $z_m({{\textbf {x}}}_i)$ in the corresponding daughter nodes. After *M* iterations from Eq. (4), the final predictor $F_M({{\textbf {x}}}_i)$ is converted into a probability estimate

6 $$\begin{aligned} \hat{p}({{\textbf {x}}}_i)=1/(1+exp(-2y_iF_M({{\textbf {x}}}_i))). \end{aligned}$$

For an outcome with *K* class labels, we encode the data into *K* “one against all” datasets with the outcomes $\{Y=k\}$ and $\{Y\ne k\}$ to compute $\hat{p}_k({{\textbf {x}}})$.

### Ensemble Algorithm with reduced dimension

The challenge of rank-based tree method is high dimensionality. When we have *p* genes, there are $O(p^2)$ calculations involved in Eq. (2) for constructing tree nodes. As a solution, we propose a two-step ensemble algorithm, in which the first ensemble step is to reduce dimensionality and the second ensemble step is to predict the outcome.

**Algorithm 2:**
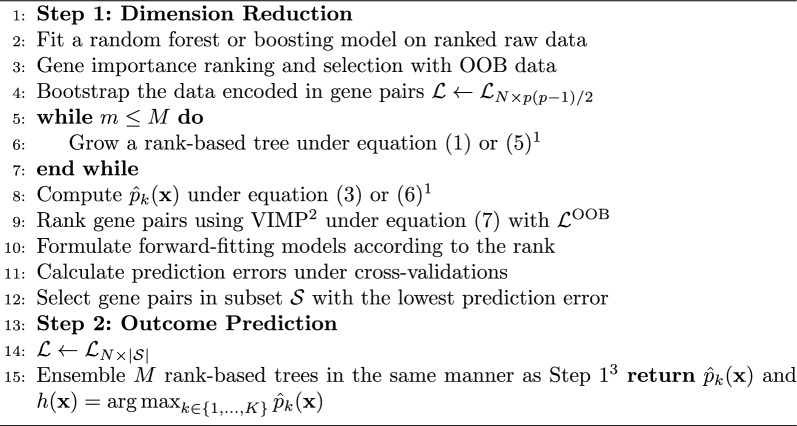
Ensemble Algorithm with Reduced Dimension

1 2 3

For variable selection, we have to construct a variable importance (VIMP) measurement based on a loss function. For classification problems, measures of performance used are the misclassification error or the Brier score [34–36]. For the latter, we have $L(Y,\hat{p})=(1/K)\sum _{k=1}^K\left( \mathbb {1}_{\{Y=k\}}-\hat{p}_k\right) ^2$. To measure VIMP, we grow each tree using a bootstrap sample of the original data and the previously mentioned OOB data is used to calculate the loss function under the original OOB data and the permuted OOB data. Let $\mathcal {L}^{\text {OOB}}$ be the OOB data and let $\hat{p}_k(\tilde{{{\textbf {x}}}}^{(ij)})$ be the estimator for permuted ${{\textbf {x}}}$ where the relationship of $X_i$ and $X_j$ is swapped in all the rank-based trees, which can be achieved by permuting the *i*th and *j*th columns in $\mathcal {L}^{\text {OOB}}$. The VIMP for gene pairs $X_i$ and $X_j$ is defined as

7 $$\begin{aligned} I(X_i,X_j)=\frac{\sum _{s \in \mathcal {L}^{\text {OOB}}}L[y_s,\hat{p}({{\textbf {x}}}_s)]}{|\mathcal {L}^{\text {OOB}}|}-\frac{\sum _{s \in \mathcal {L}^{\text {OOB}}}L[y_s,\hat{p}_k(\tilde{{{\textbf {x}}}}_s^{(ij)})]}{|\mathcal {L}^{\text {OOB}}|}. \end{aligned}$$

Utilizing VIMP, the two-step ensemble algorithm is described in Algorithm 2.

## Gene expression data and evaluation methods

In the next section, we evaluate the effectiveness of our ensemble methods of rank-based trees, as depicted in Fig. 1D, on gene expression datasets of both binary and multi-class outcomes. In this regard, we gathered 12 publicly accessible gene expression datasets, with sample sizes ranging from 22 to 587 and numbers of genes ranging from 85 to 2526. Table 1 summarizes these datasets, which are all related to studies of human cancer, including liver, central nervous system, brain, prostate, lymphoma, breast, small round blue cell tumors, leukemia, lung and bladder. Further information can be obtained by referring to the relevant publications. The last dataset studies the classification of triple negative breast cancer (TNBC) with four subtypes [37], including two basal-like (BL1 and BL2) subtypes, a mesenchymal (M) subtype, and a luminal androgen receptor (LAR) subtype. To evaluate the prediction performance of our methods in cross-platform scenarios, we also downloaded the TNBC datasets generated from RNA sequencing in [38] with a sample size of 26; in [39] with a sample size of 475; and in the Cancer Genome Atlas database [40] with a sample size of 136. The dataset in [37] was generated from the Affymetrix (Affy) GeneChip microarray; therefore, our training dataset and test dataset are from different platforms.

**Table 1:** Binary and multi-class datasets of gene expression profiles for cancer discrimination

Datasets^a^	Platform	$N$^b^	$P$ ^b^	$K$ ^b^	Class sample size	References
Liver	cDNA	180	85	2	HCC/liver = 104/76	[41]
CNS	Affy	34	857	2	CMD/DMD = 25/9	[42]
Glioblastoma	Affy	22	1152	2	CO/NO = 7/15	[43]
Prostate	Affy	77	339	2	PR/N = 58/19	[44]
NHL	cDNA	42	1095	2	DLBCL_1_/DLBCL_2_ = 21/21	[45]
Breast	Affy	49	1198	2	ER+/ER− = 25/24	[46]
SRBCTs	cDNA	83	1069	4	BL/EWS/NB/RMS = 29/11/18/25	[47]
Leukemia	Affy	72	2194	3	MLL/ALL/AML = 24/20/28	[48]
Lung	Affy	203	1543	5	ADE/SQU/SCC/NO = 139/17/6/21/20	[49]
Bladder	Affy	40	1203	3	C1/C2/NO = 9/20/11	[50]
ALL	Affy	248	2526	6	TALL/E2A/BCR/TEL/MLL/NO = 15/27/64/20/79/43	[51]
TNBC	Affy & RNAseq^c^	375	2188	4	BL1/BL2/M/LAR = 125/80/67/103	[37]

^a^*CNS* central nervous system, *AODs* anaplastic oligodendrogliomas, *NHL* Non-Hodgkin’s lymphoma, *SRBCTs* small round blue cell tumors, *ALL* acute lymphoblastic leukemia, *TNBC* triple negative breast cancer

^b^*N* stands for number of samples, *P* for number of genes and *K* for number of classes

^c^We downloaded other three datasets [38–40] and trained our models on data from one platform (e.g. microarray) while tested its prediction performance on data from another platform (e.g. RNA-seq)

### Other SSP methods and algorithm implementation

Beside the *k*-TSP method, we also compared our methods with the nearest template prediction (NTP) method, which compares the gene expression profile of a single sample to a pre-defined set of gene expression profiles, known as templates. The subclass label can be determined using a distance metric (e.g. cosine distance, Euclidean distance, etc.) as the similarity to each template [14]. In our comparison, *k*-TSP was implemented from the “switchbox” R package [52], in which the optimal number of gene pairs was selected from a range of values from 2 to 10 with fivefold cross-validation. For multi-class classification, a one-vs-one scheme was used and a classifier was trained for each pair of subclasses [53]. To avoid ties in majority voting, only odd numbers were considered during training. We implemented the NTP method with the “CMScaller” package [54], which was originally created for classifying colorectal cancer pre-clinical models [4, 55]. The prediction for each sample was determined using the sample’s closest cosine distance to each template. We utilized the “gbm” R package [56] for implementing our boosting algorithm and the “randomForestSRC” R package for our random forest algorithm [57]. For the random forest implementation, we adopted the multi-class tree with class-balanced sampling instead of fitting separate one-versus-rest models for each class [27] to improve computational efficiency and prediction performance. We noticed that there are other classical methods available, such as *k*-nearest neighbor (KNN) and support vector machines (SVM). We did not present the results in Section 4 because the comparison was already presented in Tan et al. [21] and showed that *k*-TSP works superior or comparable to KNN and SVM (see Tables 3 and 4 in Tan et al. [21], and we have the same conclusion with them). We also tried random forest/boosted trees using single gene features, the results of which are similar to SVM, and we did not include the results due to limited space.

### Performance measures

**Fig. 2:**
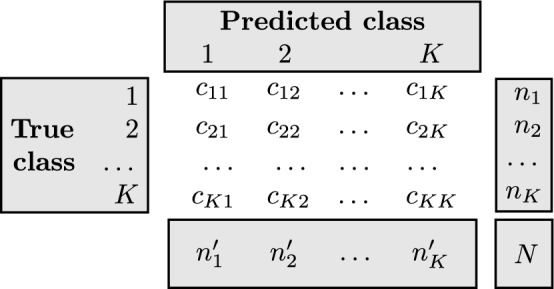
Confusion matrix for a dataset with *K* classes

Given a dataset with sample size *N* and an outcome of *K* classes, let $c_{ij}$ be the number of samples belonging to class *i* that are predicted to the *j*th class and the sample size for class *i* is denoted as $n_i=\sum _{j=1}^{K}c_{ij}$ (see Fig. 2). The performance measure is defined as accuracy (ACC):

8 $$\begin{aligned} \text {ACC}=\frac{\sum _{i=1}^{K}c_{ii}}{N}. \end{aligned}$$

Note that ACC is highly influenced by the imbalanced sample sizes among different classes. Therefore, we subsample or bootstrap the data such that $n_i/N\approx 1/K$. All datasets were randomly divided into class-balanced training (70%), validation (15%), and test data (15%). To evaluate the robustness and assess the performance of the methods, we fitted the four models on the training data, used the validation data for tuning parameters, and compared the ACC values on the corresponding test data. We replicated this procedure 50 times to compare the ACC values.

## Results

**Fig. 3:**
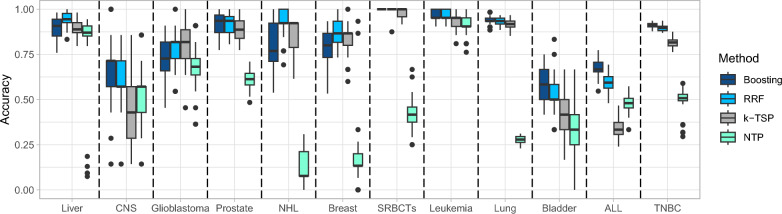
Model performance on benchmark datasets

Figure 3 summarizes the ACC results of our proposed methods, random rank forest (RRF) and boosting algorithm with the LogitBoost cost (Boosting), on the benchmark datasets in Table 1 with a comparison of the *k*-TSP and NTP methods. The accuracy values were calculated using Eq. (8). The results show that our proposed methods work comparably well and outperform the existing *k*-TSP and NTP methods. For the binary classification problems, the accuracy values from boosting, RRF, *k*-TSP and NTP are 0.89, 0.94, 0.90 and 0.80 for the Liver dataset; 0.65, 0.60, 0.47 and 0.53 for the CNS dataset; 0.73, 0.77, 0.79, 0.69 for the Glioblastoma dataset; 0.92, 0.92, 0.89 and 0.62 for the Prostate dataset; 0.80, 0.93, 0.86 and 0.13 for the NHL dataset; and 0.80, 0.88, 0.84 and 0.17 for the Breast dataset. Overall, RRF has better performance than Boosting in binary classifications. Muti-class problems are more challenging than binary classifications for all four methods, in which Boosting typically outperforms RRF. For the multi-class problems, the accuracy values from boosting, RRF, *k*-TSP and NTP are 1.00, 1.00, 0.98 and 0.42 for the SRBCTs dataset; 0.97, 0.97, 0.93 and 0.92 for the Leukemia dataset; 0.94, 0.93, 0.92, 0.28 for the Lung dataset; 0.58, 0.53, 0.41 and 0.36 for the Bladder dataset; 0.67, 0.59, 0.34 and 0.48 for the ALL dataset; and 0.91, 0.90, 0.82 and 0.50 for the TNBC dataset. The NTP method is among the weakest performance because it does not have a feature selection procedure. RRF and the boosting algorithm outperform *k*-TSP because they extend the framework of *k*-TSP from one gene-pair comparison at a time to integrating a large number of interacted gene-pair comparisons.

Both boosting and random forest have proven to be successful in our real-data applications. Their effectiveness stems from their ability to handle high-dimensional complex relationships, reduce overfitting, and provide robust predictions by leveraging ensemble methods. However, the choice between boosting and random forest depends on the specific dataset characteristics, and it is often a matter of empirical evaluation to determine which method performs better for a given task. We recommend random forest over boosting for multiclass problems and large-size datasets since the boosting model has to transform multiclass outcomes into binary outcomes to calculate loss function and trees in boosting models are sequentially grown instead of parallelly grown. The *k*-TSP outperforms the NTP method because it can be more robust to noise and outliers. By considering multiple top scoring pairs, the influence of individual noisy or outlier templates is reduced, leading to more reliable predictions. On the other hand, the NTP method is more susceptible to the influence of outliers or noise in the template set because it relies on a single nearest template.

Table 2 shows the dimension reduction results from RRF and boosting. There are two stages for variable selection: gene selection in the initial stage and gene-pair selection in the subsequent stage, whose results were displayed as the number of genes and number of gene pairs selected, respectively. For the TNBC dataset, 49 common genes are identified after data preprocessing across different platforms, which are all considered informative variables by the algorithm. Although the prediction performance of boosting and random forest appears comparable, it is an interesting observation that boosting tends to select fewer variables than random rank forests. However, the variance of the total selected variables by RRF appears to be smaller than that observed with boosting. We posit that rank-based trees excel in borrowing information across different variables, resulting in a robust prediction performance despite variations in variable selection results.

**Table 2:** Dimension reduction results from random forest and boosting

	Random Rank Forest				Boosting			
	# of genes^a^		# of Gene pairs^b^		# of genes		# of gene pairs	
	Mean^c^	SD^c^	Mean	SD	Mean	SD	Mean	SD
Liver	84.98	0.14	728.78	36.37	70.02	13.03	159.28	48.74
CNS	89.60	3.27	207.18	18.86	40.82	10.51	1.54	3.68
Glioblastoma	167.94	11.61	192.86	16.41	53.34	37.35	45.66	42.36
Prostate	253.46	10.90	622.16	44.49	126.46	62.02	112.44	65.86
NHL	200.56	12.09	200.80	20.51	53.78	50.04	19.54	27.68
Breast	231.18	15.02	256.22	25.39	50.08	35.36	33.74	35.75
SRBCTs	461.50	16.52	501.48	15.54	206.77	92.36	282.57	121.13
Leukemia	327.90	18.24	316.14	23.91	109.88	49.24	117.00	49.49
Lung	588.84	20.05	971.28	38.90	685.8	164.56	920.22	131.11
Bladder	342.88	15.18	421.02	19.29	91.84	52.94	76.90	46.89
ALL	1782.52	25.62	2923.06	59.08	379.68	104.62	407.97	150.75
TNBC	49.00	0.00	1021.98	7.79	49.00	0.00	427.84	41.63

^a^Total number of selected genes from Line 3 in Algorithm 2

^b^Total number of selected gene pairs from Line 9 in Algorithm 2

^c^Mean and standard deviation (SD) were calculated from 50 replications

As mentioned in the previous section, one advantage of RRF is its capacity to extract precise and easily understandable rules that offer biological insights into the classification process. We used the terminal nodes of rank-based trees as the candidate “simple decision rules” and adopted a similar algorithm of *k*-TSP [21] to rank and select these candidate rules. The result for the Liver dataset is listed in Table 3. These rules are different from those in the *k*-TSP methods since *k*-TSP only ranks gene pairs one by one, while rules from trees are combinations of multiple gene pairs. We found that this multivariate fashion can improve prediction accuracy with much fewer rules than *k*-TSP. The accuracy values from boosting, RRF, *k*-TSP and NTP are 0.89, 0.94, 0.90 and 0.80 for this dataset, while adopting only four rules in Table 3 could provide comparable accuracy of 0.85. The results obtained validate the findings of [41], which demonstrated that our method is capable of generating accurate and interpretable decision rules for effectively classifying microarray data.

**Table 3:** Classification rules from Algorithm 1 for the liver dataset

#	If	Then	Else
1	*NCOR1 > BNIP2* and *DEF6 < LY6E*	Liver	HCC
2	*LSM8 > NFS1* and *OLFML2B > SMAD7* and *SDF2 < MAPK14*	Liver	HCC
3	*LY6E < NMT1-PLCD3* and *BNIP2 < HPGDS*	Liver	HCC
4	*LY6E < TCF4* and *DEF6 < B3GNT5*	Liver	HCC

## Discussion

The results shown in the previous section are subject to specific tuning parameters, which are discussed in this section. Although the following results are problem-specific, they show some robustness of our model and provide some insight for the readers to customize grid search on their own. The following parameters are influential for optimizing the model’s behavior and adapting it to specific datasets. We suggest systematically exploring the parameter space, evaluating different configurations, and selecting the optimal set of parameter values based on performance metrics.

### Learning rate $\lambda$ for boosting

**Fig. 4:**
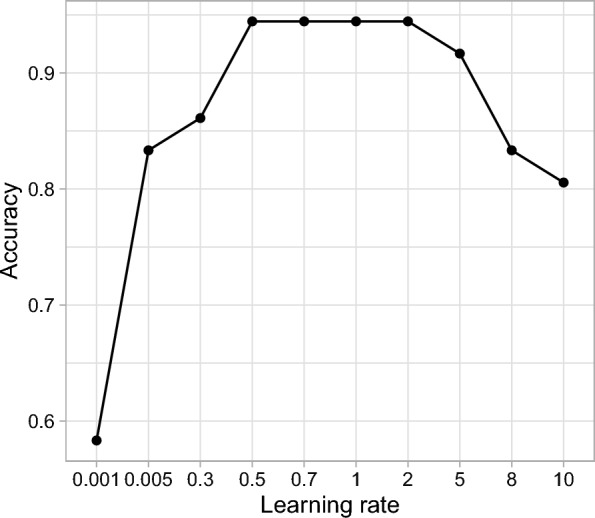
Effect of learning rate of boosting on model performance

The learning rate in boosting algorithms shown in Eq. (4) determines the contribution of each weak learner (e.g., rank-based tree) to the final ensemble model. It controls the amount by which the weights of misclassified samples are adjusted in each iteration of boosting. The learning rate influences boosting via the speed of convergence, model complexity, model accuracy and robustness to noise and outliers. Figure 4 demonstrates the effect of the learning rate on the classification of the Liver dataset. Overall, the model is robust to learning rates in a wide range. It’s important to note that the optimal learning rate for boosting depends on the specific dataset and problem at hand. We used cross-validation to determine the learning rate that achieves the best balance between convergence speed, accuracy, and robustness for a given dataset.

### Number of trees/iterations *M*

**Fig. 5:**
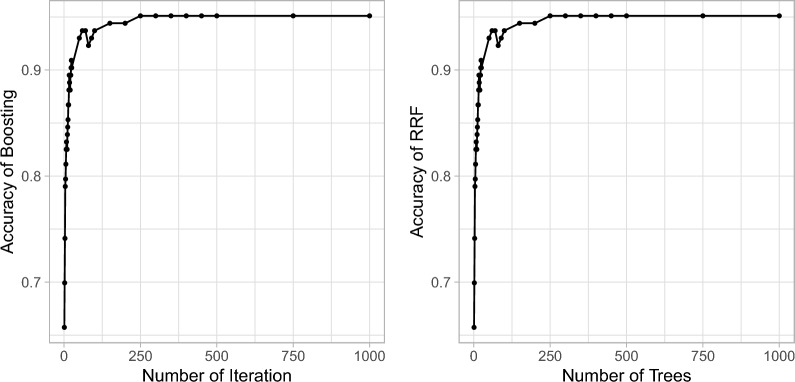
Robustness of iteration/tree number to model performance

The number of trees is an important parameter in both boosting algorithms and random forests. Increasing the number of trees tends to improve the model’s performance since as more trees are added, the boosting model can better capture complex patterns and reduce both bias and variance errors; with more trees, the random forest ensemble becomes more robust and stable as it aggregates predictions from a larger number of diverse decision trees. Figure 5 demonstrates the influence of iteration/tree number on model performance for the Liver dataset, where a number of 250 seems sufficient for both random forest and boosting. For all the datasets, adding too many trees is unlikely to increase the risk of overfitting; however, increasing the number of trees also increases the computational cost of training and inference. Therefore, there is a trade-off between model performance and computational resources. From our empirical experimentation, an iteration number of 500 is sufficient for most datasets in both random forests and boosting and increasing the number larger than 1000 is unlikely to make any difference.

### Depths of trees and terminal node size

**Fig. 6:**
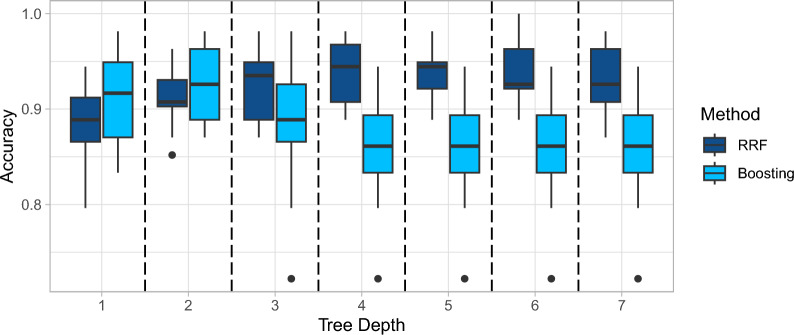
Effect of tree depth on model performance

**Fig. 7:**
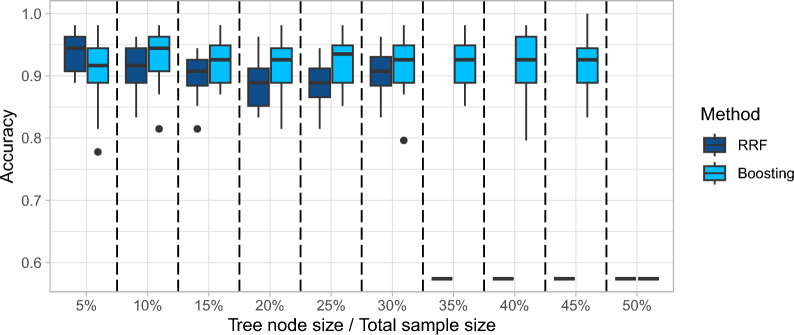
Effect of terminal node size on model performance

The depth of trees, also known as the tree’s maximum depth or tree size, plays a crucial role in growing rank-based trees. It has a similar influence as terminal node size since the deeper the tree is, the smaller the terminal node size is. In boosting algorithms, shallow trees (limited depth) are commonly used to prevent overfitting and improve the model’s generalization ability. By limiting the complexity of individual trees, boosting focuses on learning simple rules or patterns, which can be combined to form a powerful ensemble. On the other hand, random forests typically use deep trees to achieve higher accuracy and capture more complex relationships in the data. Deeper trees can capture intricate patterns and interactions among features, which can improve the model’s predictive power. Random forests overcome overfitting caused by deep trees via averaging across a large number of trees. As shown in Figs. 6 and 7 for the Liver dataset, it is crucial to strike a balance between the tree depth and the model’s generalization ability in both boosting and random forests. The optimal tree depth depends on the dataset characteristics, and we used cross-validation to determine the appropriate tree depth without a specific constraint on the terminal node size.

Note that the tree depth of 1 in the first column of Fig. 6 for random forest is roughly equivalent to the *k*-TSP method since a tree of one split is equivalent to a top scoring pair. Figure 6 demonstrates that extending the *k*-TSP method via growing deeper trees and ensemble methods can achieve higher accuracy in prediction.

### Number of competing variables *q* at each split

**Fig. 8:**
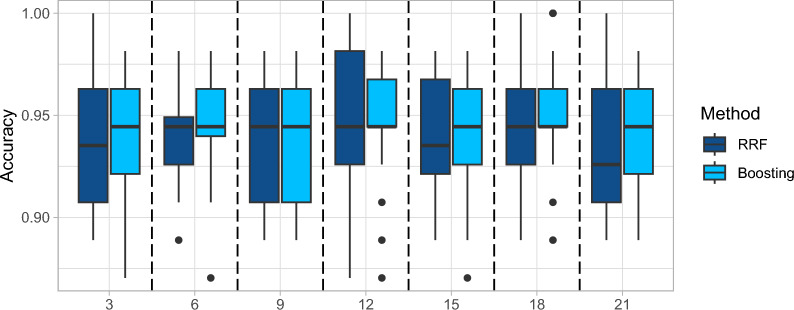
Robustness of competing ranked pairs to model performance

The number of competing gene pairs at each split, also known as feature subspace size, is defined in Eq. (2) denoted as *q*. A larger *q* will increase the computational cost. However, it does not hold much significance in boosting algorithms nor random forests. Boosting algorithms typically do not involve explicit feature subsampling at each split. Instead, they focus on sequentially adjusting the weights of training examples to improve the model’s performance. Therefore, the number of competing variables at each split does not directly impact boosting. In random forests, the number of competing variables at each split determines the randomness and diversity among decision trees in the ensemble. A smaller number of competing features at each split helps to decorrelate the trees in the random forest ensemble and prevents a few dominant features from overshadowing others. It promotes diversity among the trees, leading to a more robust and accurate ensemble. However, as shown in Fig. 8, random forest is also robust to the number of competing variables since the total number of variables is large in genetic datasets. In other words, when $q<<p$, the influence of *q* is small.

## Conclusions

In this study, we introduce an advanced rank-based tree model that builds upon TSP methods, incorporating ensemble techniques such as boosting and random forests to achieve enhanced predictive power. This approach allows us to derive interpretable rules from the terminal nodes of rank-based trees, akin to TSP methods. Our classifiers, grounded in the ranking of gene expression values within individual profiles, remain robust against preprocessing effects. When tested across twelve diverse human cancer gene expression datasets, both binary and multi-class, our methods demonstrated marked superiority over traditional k-TSP and NTP classifiers. A notable feature of our Random Forest-derived rules is their succinctness, comprising fewer gene pairs while maintaining or surpassing accuracy in predictions.

The strength of our approach lies in the multivariate capability of decision trees, which adeptly adjust for multiple ranked gene pairings. This ability to encapsulate intricate gene-target outcome relationships enables the learning of complex non-linear patterns and gene interactions. In contrast, conventional TSP methods, often restricted to basic if-then logic, may falter in capturing these complexities. Our method addresses the common issue of overfitting in tree models by integrating ensemble techniques, which enhances both the accuracy and robustness of the predictions. This integration avoids the complexities of tree construction rules, focusing instead on leveraging the collective strength of multiple decision trees [58].

Furthermore, these rank-based trees serve as fundamental units in ensemble methods, such as random forests and boosting algorithms. The aggregating of multiple trees in these methods not only improves prediction accuracy but also offers resilience against model biases. By employing data resampling techniques, we utilize class-balanced sampling strategies, effectively addressing the prevalent challenge of class imbalance in many datasets [27, 59–61]. This approach offers a notable advantage over the one-versus-rest models, which, despite their appearance of treating class categories equally, still grapple with class imbalance within individual category models.

While tree-based algorithms offer optimization avenues, such as missing data imputation or feature importance analysis [62], our study also acknowledges certain limitations that warrant further exploration. One such area is the handling of ties in ranking variables. Our methods demonstrated reduced effectiveness in datasets with abundant zero values, suggesting the need for strategies like introducing artificial noise to enhance model performance [63]. Another aspect for future refinement is the computational intensity of our dimension reduction step, which currently relies on random forest or boosting models, as opposed to more straightforward filter methods [64]. Addressing these limitations will be pivotal in our ongoing efforts to refine and enhance the efficacy of rank-based tree methods for gene expression data classification.

## Data Availability

We used publicly available data for this research. The data and source code can be downloaded from: https://github.com/TransBioInfoLab/ranktreeEnsemble.

## Abbreviations

SSP: Single Sample Predictor
TSP: Top Scoring Pairs
TNBC: Triple negative breast cancer
LAR: Luminal androgen receptor
CNS: Central nervous system
AODs: Anaplastic oligodendrogliomas
NHL: Non-Hodgkin’s lymphoma
SRBCTs: Small round blue cell tumors
ALL: Acute lymphoblastic leukemia
NTP: Nearest template prediction
KNN: *k*-nearest neighbor
SVM: Support vector machines
ACC: Accuracy
RRF: Random Rank Forest

## Footnotes

1 Eqs. (1) and (3) are for random rank forest, while Eqs. (5) and (6) are for boosting.

2 We assigned cases to the opposite split when comparing “less than or equal to” and “greater than” for the corresponding gene pairs instead of permuting the raw data.

3 For random forest, OOB data is used for prediction.
